# Early afterdepolarizations in cardiac action potentials as mixed mode oscillations due to a folded node singularity

**DOI:** 10.1371/journal.pone.0209498

**Published:** 2018-12-31

**Authors:** Philipp Kügler, André H. Erhardt, M. A. K. Bulelzai

**Affiliations:** 1 Institute of Applied Mathematics and Statistics, University of Hohenheim, Stuttgart, Germany; 2 Department of Mathematics, University of Oslo, Oslo, Norway; 3 Departmeny of Basic Sciences and Related Studies, Quaid-e-Awam University of Engineering, Science and Technology, Nawabshah, Pakistan; Georgia State University, UNITED STATES

## Abstract

Early afterdepolarizations (EADs) are pathological voltage oscillations during the repolarization phase of cardiac action potentials. They are considered as potential precursors to cardiac arrhythmias and have recently gained much attention in the context of preclinical drug safety testing under the Comprehensive in vitro Proarrhythmia Assay (CiPA) paradigm. From the viewpoint of multiple time scales theory, the onset of EADs has previously been studied by means of mathematical action potential models with one slow ion channel gating variable. In this article, we for the first time associate EADs with mixed mode oscillations in dynamical systems with two slow gating variables and present a folded node singularity of the slow flow as a novel mechanism for EADs genesis. We derive regions of the pharmacology parameter space in which EADs occur using both the folded node analysis and a full system bifurcation analysis, and we suggest the normal distance to the boundary of the EADs region as a mechanism-based risk metric to computationally estimate a drug’s proarrhythmic liability.

## Introduction

Early afterdepolarizations (EADs) are abnormal depolarizations during the repolarization phase of the cardiac action potential and may be caused by drugs, oxidative stress or ion channelopathies. EADs are an important cause of cardiac arrhythmias such as polymorphic ventricular tachycardia (PVT) or torsades de pointes (TdP) [1], [2]. Recently, EADs propensity, expressed in terms of the net charge carried by major ionic currents during an action potential, has been chosen as an in-silico biomarker [3] for TdP risk evaluation of drugs within the CiPA (Comprehensive in vitro Proarrhythmia Assay) initiative [4], [5], [6] to overhaul the current cardiac drug safety regulations.

Computational models of cardiac cells [7] form the basis for the mathematical analysis of EADs. The most common approach is to numerically simulate single cell action potential models after on purpose or random model parameter variations such that EADs occur [8], [9], [3], [10], [11], [12]. Numerical simulation studies have also been performed at the cardiac tissue and cardiac organ level in order to analyze the synchronization of EADs and the triggering of arrhythmic patterns due to EADs [13], [14], [15]. Furthermore, numerical continuation has been used in [16] to explore the bifurcation structure of non-paced human ventricular myocyte models and to relate it to parameter regions of EADs occurence in the periodically paced model counterparts. EADs have also been investigated by bifurcation analysis applied to parametrized families of fast subsystems that are obtained from separating a *single slow variable*, related to ion channel gating, from the full cell model: while in [17], [18], [19], the onset of EADs has been linked to the existence of a supercritical Hopf bifurcation in the fast subsystem, it was shown in [20] that EADs may also arise in the presence of a subcritical Hopf bifurcation as well as in the complete absence of Hopf bifurcations in the fast subsystem.

**Fig 1:**
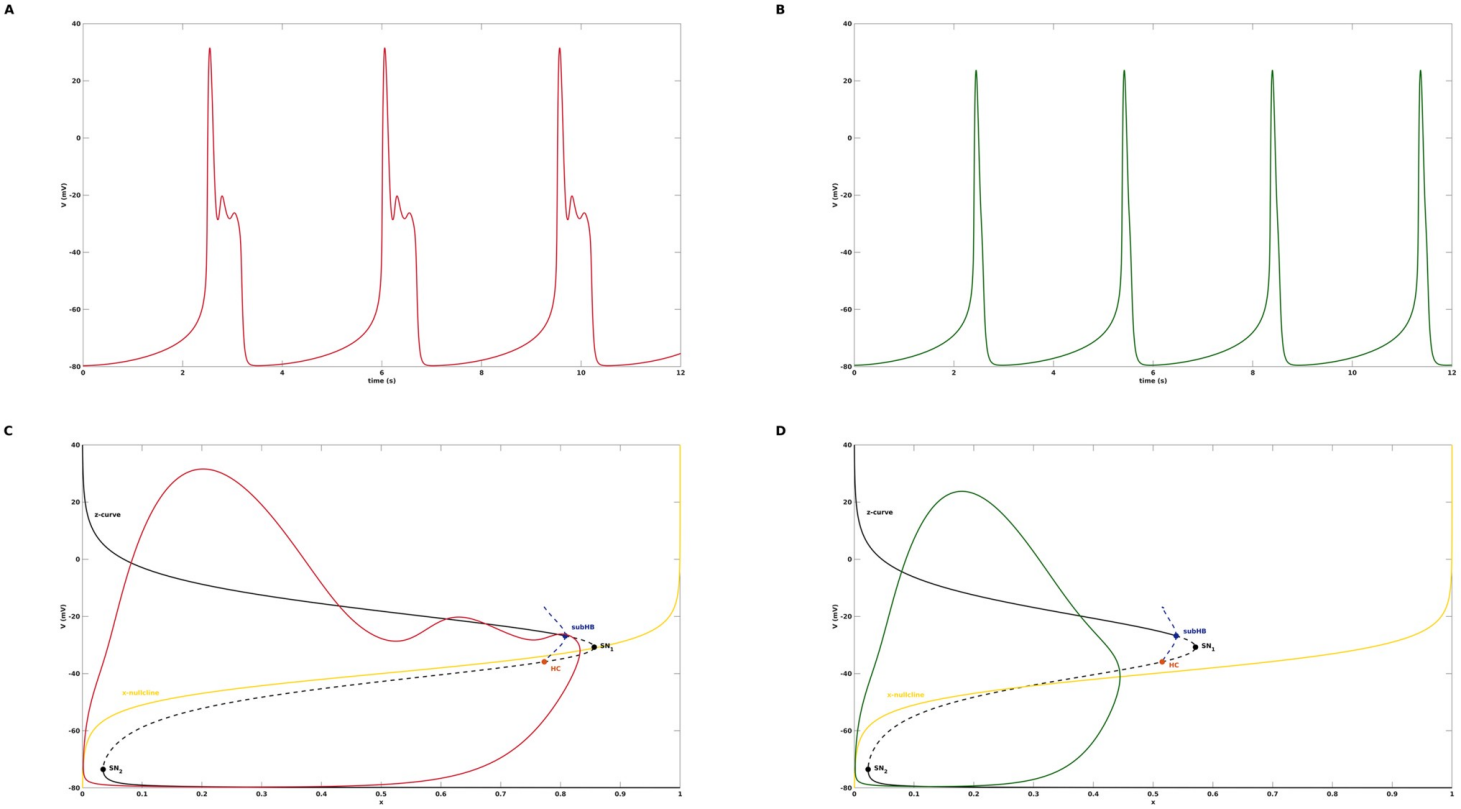
Failure of standard fast-slow analysis to clarify disappearance of EADs. A: Action potential with EADs in the form of a 1^2^ mixed mode oscillation, obtained with the default parameter setting from Table 1. B: Action potential with normal repolarization in the form of a 1^0^ mixed mode oscillation, obtained after changing *G*_*K*_ from 0.04 to 0.06. C: Standard fast-slow analysis for the default parameter setting. The bifurcation diagram for (2) with *G*_*K*_ as continuation parameter contains a z-curve with three branches that are connected via two saddle node bifurcations SN_2_ and SN_1_: a bottom branch of stable equilibria (solid line) of (2), a middle branch of unstable equilibria (dashed line), and a top branch of both stable and unstable equilibria of (2). At the top branch, the stability changes at a subcritical Hopf bifurcation *subHB* from which unstable limit cycles emerge that terminate at a saddle-homoclinic bifurcation *HC*. The blue dashed lines denote the maximum and minimum voltage values of the unstable limit cycles. The red line shows the projection of the 1^2^-trajectories from A onto the bifurcation diagrams, the yellow line is the nullcline of the variable *x*. D: Standard fast-slow analysis for *G*_*K*_ = 0.06. The arrangement of the z-curve, the subcritical Hopf bifurcation and the x-nullcline is similar to C and does not explain why the EADs disappear in the transition from *G*_*K*_ = 0.04 to *G*_*K*_ = 0.06.

In this study, we consider action potentials with EADs as mixed mode oscillations (MMOs), see [21] for a review, of signature 1^*s*^ that are comprised of 1 large amplitude oscillation and *s* small amplitude oscillations. As an example, see Fig 1A for a 1^2^ pattern corresponding to an action potential (AP) distorted by EADs and Fig 1B for a 1^0^ pattern representing an undisturbed AP. We again take advantage of the difference in time scales between the dynamic variables of a cardiac cell model but apply—as opposed to previous multiple time scale investigations of EADs—a fast/slow analysis technique that features *two slow variables*. We demonstrate that the corresponding slow flow, which is restricted to a surface called critical manifold, admits a folded node singularity characterized by two real eigenvalues of the Jacobian that have the same sign. Then, the theory of MMOs [22], [23] implies that the trajectories of the full system are organized, locally near the folded node, by twisted slow manifolds [24]. This twisting organizes the small amplitude oscillations which in combination with a global return mechanism determined by the critical manifold gives rise to the MMO patterns that underlie cardiac action potentials with EADs.

MMOs due to a folded node have been previously discussed, e.g., in the context of electrical bursting in pituitary cells [25], [26], [27] or climate change models [28], [29]. However, to the best of our knowledge, this study is the first that connects this particular MMO mechanism with EADs in cardiac action potentials and hence makes a novel contribution to EADs analysis. Furthermore, the conditions for the occurrence of such MMOs due to a folded node, namely existence of a folded node singularity and return of the large amplitude oscillation to the basin of attraction of the small amplitude oscillatory state, define borders of the EADs region in the model parameter space, which—further away from the singular limit—may be adapted by a complementary bifurcation analysis of the full system. This allows us to introduce the normal distance of a pharmaceutical compound to these EADs boundaries as a mechanism-based proarrhythmic risk metric, which may enrich the current discussion of computational biomarkers for the classification of a drug’s proarrhythmic liability within the CiPA initiative.

## Materials and methods

### Cardiac action potential model

Computational models of cardiac action potentials describe the dynamics of the transmembrane voltage *V* in dependence of the ionic currents and, for more than 50 years [30], [31] are an important tool in studying cardiac electrophysiology. Modern cardiac AP models [32], [9], [3], [12] consist of dozens of state variables and hundreds of model parameters, a complexity that results from the tremendous insight gained under the holistic paradigm of systems physiology but often hampers model analysis and validation. An alternative approach is to use parsimonious cardiac AP models [33], [34], [35], [36] that are low dimensional and only permit to address one or two phenomena but are amenable to mathematical analysis that goes beyond pure numerical simulation. In the context of EADs analysis, the model

$$\begin{array}{cccc} C_{m}\frac{dV}{dt} & =-G_{Ca}d_{\infty }\left(V\right)f\left(V-E_{Ca}\right)-G_{K}x\left(V-E_{K}\right) & & =:h(V,f,x),\\ \frac{df}{dt} & =\frac{f_{\infty }\left(V\right)-f}{\tau_{f}} & & =:g_{1}\left(V,f,x\right),\\ \frac{dx}{dt} & =\frac{x_{\infty }\left(V\right)-x}{\tau_{x}} & & =:g_{2}\left(V,f,x\right), \end{array}$$ (1)

has been introduded in [37] and subsequently also used in [38], [20]. In particular, this ODE model of state dimension *n* = 3 features one inward calcium current

$$\begin{array}{c} I_{Ca}=G_{Ca}d_{\infty }\left(V\right)f\left(V-E_{Ca}\right) \end{array}$$

with the calcium channel conductance *G*_*Ca*_ and the dynamic inactivation variable *f*, as well as one outward potassium current

$$\begin{array}{c} I_{K}=G_{K}x\left(V-E_{K}\right) \end{array}$$

with the potassium channel conductance *G*_*K*_ and the dynamic activation variable *x*. The corresponding steady state variables are given by

$$\begin{array}{ccc} d_{\infty }\left(V\right) & = & \frac{1}{1+e^{\frac{V-VT_{d}}{k_{d}}}},\\ f_{\infty }\left(V\right) & = & \frac{1}{1+e^{\frac{V-VT_{f}}{k_{f}}}},\\ x_{\infty }\left(V\right) & = & \frac{1}{1+e^{\frac{V-VT_{x}}{k_{x}}}}. \end{array}$$

The default parameter values from [37] are given in Table 1 and correspond to an action potential with EADs in form of a 1^2^ mixed mode oscillation, see Fig 1.

**Table 1:** Default parameter values.

*C*_*m*_	*E*_*Ca*_	*G*_*Ca*_	*E*_*K*_	*G*_*K*_	*VT*_*f*_	*k*_*f*_	*τ*_*f*_	*VT*_*x*_	*k*_*x*_	*τ*_*x*_	*VT*_*d*_	*k*_*d*_
1	100	0.025	-80	0.04	-20	8.6	80	-40	-5	300	-35	-6.24
*μ*F/cm^2^	mV	mS/cm^2^	mV	mS/cm^2^	mV	mV	ms	mV	mV	ms	mV	-mV

### Numerical methods

The model Eq (1) (and all of its subsystems, see the following subsections) were written in Matlab R2017b [39]. Initial value problems were integrated using the ode15s solver with relative and absolute error tolerances of 1e-10 and analytically derived Jacobian matrix, boundary value problems were solved using the bvp4c solver. Numerical curve continuation and bifurcation analysis was performed using both MATCONT 6.10 [40] and AUTO-07P [41].

### Preliminaries for standard fast-slow analysis

The state variables of cardiac action potential models typically change at different time scales. With respect to the model (1) and the default parameter setting from Table 1, the time constants of the variables *f* and *x* are given by *τ*_*f*_ = 80 and *τ*_*x*_ = 300, respectively. The time constant of the variable *V* can be estimated as $\tau_{V}=\frac{C_{m}}{G_{Ca}+G_{K}}=15.38$. Hence, *τ*_*V*_ < *τ*_*f*_ < *τ*_*x*_, such that *x* is the slowest and *V* is the fastest variable.

The standard approach of studying EADs by multiple time scale analysis [17], [18], [20], [19] is to separate a single variable—namely the slowest one—from the faster ones. In case of (1), this yields a (2, 1)-fast-slow system with the fast subsystem

$$\begin{array}{ccc} C_{m}\frac{dV}{d\tau } & = & h(V,f,x),\\ \frac{df}{d\tau } & = & g_{1}\left(V,f,x\right),\\ \frac{dx}{d\tau } & = & 0, \end{array}$$ (2)

where *τ* denotes the fast time scale. Hence, the slow variable *x* acts as a constant model parameter in the equations for the fast variables *V* and *f*. Formally, the fast subsystem (2) is obtained by taking the singular limit *τ*_*x*_ → ∞ in (1).

### Preliminaries for (1, 2)-fast-slow analysis

An alternative to the standard fast-slow approach is to treat (1) as a (1, 2)-fast-slow system, in which not only *x* but also *f* is considered as a slow variable. Taking the singular limit *C*_*m*_ → 0, the trajectories of (1) converge during fast episodes to solutions of the fast subsystem or the layer equations

$$\begin{array}{ccc} \frac{dV}{d\tau } & = & h(V,f,x),\\ \frac{df}{d\tau } & = & 0,\\ \frac{dx}{d\tau } & = & 0, \end{array}$$ (3)

where *τ* = *t*/*C*_*m*_ is the fast time scale. The equilibrium set of the system (3) is called the critical manifold

$$\begin{array}{c} C_{0}=\{(V,f,x)\;|\;h(V,f,x)=0\}. \end{array}$$ (4)

During slow episodes, the trajectories of (1) rather converge to solutions of the slow flow or the reduced system

$$\begin{array}{ccc} 0 & = & h(V,f,x),\\ \frac{df}{dt} & = & g_{1}\left(V,f,x\right),\\ \frac{dx}{dt} & = & g_{2}\left(V,f,x\right). \end{array}$$ (5)

Hence, the slow flow is described by a differential algebraic system whose phase space is given by the critical manifold (4).

**Fig 2:**
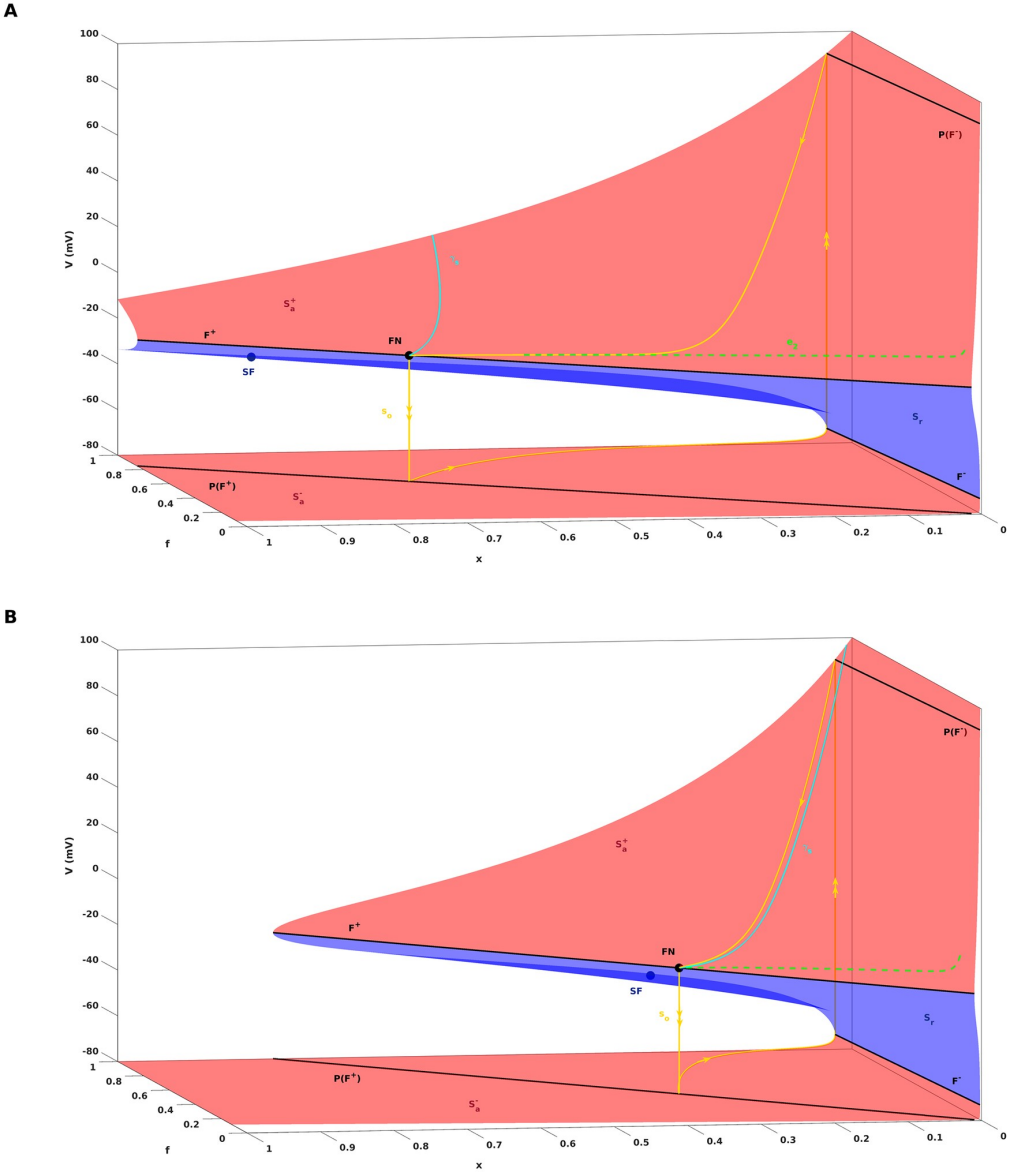
Critical manifold *C*_0_ and singular periodic orbit *s*_*o*_. The figure shows the critical manifold (4) associated with the cardiac AP model (1) for two different values of the model parameter *G*_*K*_. *C*_0_ is a folded surface in $R^{3}$ with attracting $S_{a}^{+}$, $S_{a}^{-}$ and repelling sheets *S*_*r*_ separated by two fold lines *F*^+^ and *F*^−^. The projections of *F*^+^ and *F*^−^ onto $S_{a}^{-}$ and $S_{a}^{+}$ are denoted by *P*(*F*^+^) and *P*(*F*^−^). Also shown is the singular orbit *s*_*o*_ that consists of slow segments on $S_{a}^{+}$, $S_{a}^{-}$ and fast jumps between them. Inside the funnel region, which is bounded by the singular strong canard *γ*_*s*_ and *F*^+^, all trajectories approach the folded node FN allong the eigendirection *e*_2_ associated with the singular weak canard *γ*_*w*_. SF denotes a saddle-focus equilibrium of the full system (1). A: *G*_*K*_ = 0.04. The singular orbit is projected into the funnel, which is a key reason for the appearance of EADs in the solution of the full system (1). B: *G*_*K*_ = 0.06. the singular orbit *s*_*o*_ lands outside of the funnel. Consequently, EADs do not appear in the solution of the full system (1).

Fig 2 gives an example of the critical manifold (4) for the AP model (1), and shows that, for the default parameter setting of Table 1, it is a folded surface with respect to the fast variable *V*. It consists of three sheets $S_{a}^{+}$, *S*_*r*_, $S_{a}^{-}$ separated by two disjoint fold curves *F*^+^, *F*^−^, i.e.,

$$\begin{array}{c} C_{0}=S_{a}^{+}\cup F^{+}\cup S_{r}\cup F^{-}\cup S_{a}^{-}. \end{array}$$ (6)

Here, $S_{a}^{+}$ and $S_{a}^{-}$ are the attracting upper and lower sheets of *C*_0_ that are formed by stable hyperpolarized and stable depolarized steady states of (3). While $\frac{\partial h}{\partial V}<0$ for all points on $S_{a}^{+}$ or $S_{a}^{-}$, the repelling sheet *S*_*r*_ is characterized by $\frac{\partial h}{\partial V}>0$ which corresponds to the unstable steady states of (3). Furthermore, the fold curves *F*^+^ and *F*^−^ consist of all points on *C*_0_ with $\frac{\partial h}{\partial V}=0$ that satisfy a nondegeneracy condition $\frac{\partial^{2}h}{\partial V^{2}}\neq 0$, i.e.,

$$\begin{array}{c} F^{\pm }=\{\left(V,f,x\right)\;|\;h\left(V,f,x\right)=0,\;\frac{\partial h}{\partial V}\left(V,f,x\right)=0,\;\frac{\partial^{2}h}{\partial V^{2}}\left(V,f,x\right)\neq 0\}. \end{array}$$ (7)

Taking the total time derivative of the constraint *h*(*V*, *f*, *x*) = 0, i.e,

$$\begin{array}{c} \frac{\partial h}{\partial V}\frac{dV}{dt}+\frac{\partial h}{\partial f}g_{1}+\frac{\partial h}{\partial x}g_{2}=0, \end{array}$$

the slow flow (5) can also be described by

$$\begin{array}{ccc} -\frac{\partial h}{\partial V}\frac{dV}{dt} & = & \frac{\partial h}{\partial f}g_{1}+\frac{\partial h}{\partial x}g_{2}\\ \frac{dx}{dt} & = & g_{2} \end{array}$$ (8)

restricted to *C*_0_. The system (8) is singular at the fold curves *F*^+^, *F*^−^, which means that the velocity of a trajectory goes to infinity as it approaches *F*^+^ or *F*^−^. The removal of the singularity by rescaling time according to $t_{d}=-{\left(\frac{\partial h}{\partial V}\right)}^{-1}t$ leads to the desingularized system

$$\begin{array}{ccc} \frac{dV}{dt_{d}} & = & \frac{\partial h}{\partial f}g_{1}+\frac{\partial h}{\partial x}g_{2}\\ \frac{dx}{dt_{d}} & = & -\frac{\partial h}{\partial V}g_{2} \end{array}$$ (9)

restricted to *C*_0_. The flow of (9) is equivalent to that of (8) on $S_{a}^{+}$ and $S_{a}^{-}$ but is reversed on $S_{r}^{+}$.

The equilibrium points of the desingularized flow (9) are classified into ordinary singularities defined by

$$\begin{array}{c} h=0,\;g_{1}=0\;\;\text{and}\;\;g_{2}=0 \end{array}$$

(then also equilibrium points of the full system (1)) and into folded singularities that lie on a fold curve and satisfy

$$\begin{array}{c} \frac{\partial h}{\partial f}g_{1}+\frac{\partial h}{\partial x}g_{2}=0. \end{array}$$ (10)

Given a folded singularity p_*_ = (V_*_, x_*_), the eigenvalues λ_1_, λ_2_ of the Jacobian of (9) evaluated at p_*_ decide whether p_*_ is a folded node ($\lambda_{1},\lambda_{2}\in R$ with λ_1_ ⋅ λ_2_ > 0), a folded saddle ($\lambda_{1},\lambda_{2}\in R$ with λ_1_ ⋅ λ_2_ < 0) or a folded focus ($\lambda_{1},\lambda_{2}\in C$ with $\bar{\lambda_{1}}=\lambda_{2}$).

A key observation of this study, see the Results section, is that the desingularized slow flow associated with the cardiac action potential model (1) in its default parameter setting features a folded singularity of the folded node type.

## Results

### Failure of standard fast-slow analysis

A simulation of the cardiac model (1) with the default parameter values from Table 1 yields an action potential with EADs in form of a 1^2^ mixed mode oscillation, see Fig 1. When the potassium channel conductance *G*_*K*_ is increased from 0.04 to 0.06, the EADs disappear and a normal action potential corresponding to a 1^0^ oscillatory pattern is obtained. In a first attempt to understand the disappearance of the EADs we performed a standard fast-slow analysis [17], [18], [20], [19] and studied the bifurcations of the fast subsystem (2) with the slow variable *x* as numerical continuation parameter. The bifurcation diagram, see Fig 1, features a z-curve with three branches that are connected via two saddle node bifurcations SN_2_ and SN_1_: a bottom branch of stable equilibria (solid line) of (2), a middle branch of unstable equilibria (dashed line), and a top branch of both stable and unstable equilibria of (2). At the top branch, the stability changes at a subcritical Hopf bifurcation *subHB* from which unstable limit cycles emerge that terminate at a saddle-homoclinic bifurcation *HC*. The blue dashed lines denote the maximum and minimum voltage values of the unstable limit cycles. Next, we projected the 1^2^- and the 1^0^-trajectories onto the corresponding bifurcation diagrams and complemented the latter by the nullcline of the variable *x* (solid yellow line), which is defined by *g*_2_(*V*, *f*, *x*) = 0 or *x* = *x*_∞_(*V*), respectively. Above the x-nullcline, the trajectories move to the right due to *dx*/*dt* > 0, below, they move to the left due to *dx*/*dt* < 0 until they pass *SN*_2_ and are rejected, but in total, they only roughly follow the z-curve. Both for *G*_*K*_ = 0.04 and *G*_*K*_ = 0.06, the stable equilibria at the top branch are of the focus type, i.e., the eigenvalues are conjugate-complex with negative real part, but only for *G*_*K*_ = 0.04 the trajectory feels the attraction of the foci and is forced into a transient spiraliform movement that gives rise to the EADs. While both for *G*_*K*_ = 0.04 and *G*_*K*_ = 0.06 the x-nullcline crosses the z-curve in vicinity of SN_1_ to generate an unstable equilibrium of the full system (1), only for *G*_*K*_ = 0.04, the x-nullcline also intersects the branch of unstable limit cycles, which produces an unstable limit cycle of the the full system (1). Still, the EADs start at *x*-values much lower than that of this intersection such that based on Fig 1 the latter is not involved in the EADs genesis. Overall, the arrangement of the *z*-curve, the subcritical Hopf bifurcation and the *x*-nullcline is similar for *G*_*K*_ = 0.04 and *G*_*K*_ = 0.06, such that the standard fast-slow analysis fails to explain the destruction of the EADs in the transition from *G*_*K*_ = 0.04 to *G*_*K*_ = 0.06.

### (1, 2)-fast-slow analysis reveals EADs genesis due to a folded node singularity

As an alternative to the standard fast-slow analysis, we next related the intermediate variable *f* to the slow variable *x* and determined the key objects of the resulting (1, 2)-fast-slow analysis.

#### Critical manifold, layer problem and desingularized system

The equation *h* = 0 that defines the critical manifold (4) is linear in *f* and can be solved for *f* in dependence on *V* and *x*, hence

$$\begin{array}{c} C_{0}=\{\left(V,f,x\right)\;|\;f=f\left(V,x\right)=-\frac{G_{K}x\left(V-E_{K}\right)}{G_{Ca}d_{\infty }\left(V\right)\left(V-E_{Ca}\right)}\}. \end{array}$$ (11)

For the default parameter setting from Table 1, (11) corresponds to a folded surface in $R^{3}$ that consists of two attracting sheets $S_{a}^{+}$, $S_{a}^{-}$ and one repelling sheet *S*_*r*_, see Fig 2. Note that the shape of *C*_0_ does not qualitatively change if *G*_*K*_ is set to 0.06, as *G*_*K*_ only acts as a scaling factor in (11). With respect to the fold curves *F*^+^ and *F*^−^ from (7), we considered

$$\begin{array}{ccc} \frac{\partial h}{\partial V}{|}_{f=f(V,x)} & = & -G_{Ca}\frac{d_{\infty }\left(V\right)}{dV}f\left(V,x\right)\left(V-E_{Ca}\right)-G_{Ca}d_{\infty }\left(V\right)f\left(V,x\right)-G_{K}x\\ & = & G_{Ca}d_{\infty }\left(V\right)f\left(V,x\right)[d_{\infty }\left(V\right)\left(V-E_{Ca}\right)\frac{1}{k_{d}}e^{\frac{V-VT_{d}}{k_{d}}}-1]-G_{K}x\\ & = & -\frac{G_{K}x\left(V-E_{K}\right)}{\left(V-E_{Ca}\right)}[d_{\infty }\left(V\right)\left(V-E_{Ca}\right)\frac{1}{k_{d}}e^{\frac{V-VT_{d}}{k_{d}}}-1]-G_{K}x\\ & = & -G_{K}x(d_{\infty }\left(V\right)\left(V-E_{K}\right)\frac{1}{k_{d}}e^{\frac{V-VT_{d}}{k_{d}}}+\frac{E_{K}-E_{Ca}}{V-E_{Ca}}). \end{array}$$

Consequently, the constraint

$$\begin{array}{c} \frac{\partial h}{\partial V}{|}_{f=f(V,x)}=0 \end{array}$$

is satisfied if *x* = 0 or if

$$\begin{array}{c} \frac{E_{K}-E_{Ca}}{V-E_{Ca}}=-d_{\infty }\left(V\right)\left(V-E_{K}\right)\frac{1}{k_{d}}e^{\frac{V-VT_{d}}{k_{d}}}. \end{array}$$ (12)

As for *x* = 0 the nondegeneracy condition $\frac{\partial^{2}h}{\partial V^{2}}{|}_{f=f(V,x)}\neq 0$ is violated, we concentrated on (12), which is independent of *G*_*K*_, and obtained the two solutions

$$\begin{array}{c} V^{+}\approx -24.7923\;\text{and}\;V^{-}\approx -73.5132 \end{array}$$ (13)

for the default parameter setting. Hence, the two fold curves are given by

$$\begin{array}{ccc} F^{+} & = & \{(V^{+},-\frac{G_{K}x\left(V^{+}-E_{K}\right)}{G_{Ca}d_{\infty }\left(V^{+}\right)\left(V^{+}-E_{Ca}\right)},x)\;|\;x\in \left(0,1\right]\},\\ F^{-} & = & \{(V^{-},-\frac{G_{K}x\left(V^{-}-E_{K}\right)}{G_{Ca}d_{\infty }\left(V^{-}\right)\left(V^{-}-E_{Ca}\right)},x)\;|\;x\in \left(0,1\right]\}. \end{array}$$

Fig 2 shows how *F*^+^ and *F*^−^ organize the partitioning (6) of *C*_0_ into attracting and repelling sheets.

The flow on the attracting sheets $S_{a}^{+}$, $S_{a}^{-}$ is described by the desingularized system

$$\begin{array}{ccc} \frac{dV}{dt_{d}} & = & -G_{Ca}d_{\infty }\left(V\right)\left(V-E_{Ca}\right)\frac{f_{\infty }\left(V\right)-f\left(V,x\right)}{\tau_{f}}-G_{K}\left(V-E_{K}\right)\frac{x_{\infty }\left(V\right)-x}{\tau_{x}}\\ \frac{dx}{dt_{d}} & = & G_{K}x(d_{\infty }\left(V\right)\left(V-E_{K}\right)\frac{1}{k_{d}}e^{\frac{V-VT_{d}}{k_{d}}}+\frac{E_{K}-E_{Ca}}{V-E_{Ca}})\frac{x_{\infty }\left(V\right)-x}{\tau_{x}}, \end{array}$$ (14)

while transitions from one attracting sheet to another are described by the layer problem

$$\begin{array}{ccc} \frac{dV}{d\tau } & = & -G_{Ca}d_{\infty }\left(V\right)f\left(V-E_{Ca}\right)-G_{K}x\left(V-E_{K}\right),\\ \frac{df}{d\tau } & = & 0,\\ \frac{dx}{d\tau } & = & 0. \end{array}$$ (15)

In particular, trajectories of (15) that start on the fold curves *F*^+^ and *F*^−^ land on the corresponding projections *P*(*F*^+^) and *P*(*F*^−^), see Fig 2.

#### Folded node singularity, funnel and singular periodic orbit

Next, we focused on the the folded singularities of the desingularized system (14) and constrained the defining Eq (10) to *F*^±^. This led to

$$\begin{array}{c} -G_{Ca}d_{\infty }\left(V^{\pm }\right)\left(V^{\pm }-E_{Ca}\right)\frac{f_{\infty }\left(V^{\pm }\right)-f\left(V^{\pm },x\right)}{\tau_{f}}-G_{K}\left(V^{\pm }-E_{K}\right)\frac{x_{\infty }\left(V^{\pm }\right)-x}{\tau_{x}}=0 \end{array}$$

with *V*^+^ and *V*^−^ given by (13) and *f*(*V*, *x*) defined in (11). Solving for *x*, we obtained

$$\begin{array}{ccc} x^{\pm } & = & \frac{\tau_{f}}{\tau_{f}-\tau_{x}}x_{\infty }\left(V^{\pm }\right)+\frac{G_{Ca}d_{\infty }\left(V^{\pm }\right)\left(V^{\pm }-E_{Ca}\right)}{G_{K}\left(V^{\pm }-E_{K}\right)}\frac{\tau_{x}}{\tau_{f}-\tau_{x}}f_{\infty }\left(V^{\pm }\right) \end{array}$$

and consequently two folded singularities

$$\begin{array}{c} FS^{+}=\left(V^{+},f\left(V^{+},x^{+}\right),x^{+}\right)\;\;\text{and}\;FS^{-}=\left(V^{-},f\left(V^{-},x^{-}\right),x^{-}\right). \end{array}$$

An evaluation of the Jacobian matrix of (14) at (*V*^+^, *x*^+^) and (*V*^−^, *x*^−^) showed that *FS*^+^ is a folded node singularity FN both for the default parameter values and the setting *G*_*K*_ = 0.06 due to $\lambda_{1},\lambda_{2}\in R$ with λ_1_ ⋅ λ_2_ > 0, see Table 2, while *FS*^−^ is a folded focus that lies outside of the physiological range due to *f*(*V*^−^, *x*^−^) > 1.

**Table 2:** Folded singularities of the desingularized system.

*G*_*K*_	*FS*^+^	*J*(*V*^+^, *x*^+^)	λ_1_	λ_2_	*FS*^−^
0.04	(−24.79, 0.57, 0.68)	$\left(\begin{array}{cc} -9.43\cdot 10^{-4} & -2.02\cdot 10^{-2}\\ 4.47\cdot 10^{-6} & -2.7\cdot 10^{-20} \end{array}\right)$	−8.34 ⋅ 10^−4^	−1.08 ⋅ 10^−4^	(−73.51, 1.35, 0.05)
0.06	(−24.79, 0.43, 0.34)	$\left(\begin{array}{cc} -9.75\cdot 10^{-4} & -3.04\cdot 10^{-2}\\ 7.44\cdot 10^{-6} & 2.79\cdot 10^{-20} \end{array}\right)$	−5.96 ⋅ 10^−4^	−3.79 ⋅ 10^−4^	(−73.51, 1.34, 0.03)

Both for *G*_*K*_ = 0.04 and *G*_*K*_ = 0.06, the singularity *FS*^+^ is a folded node due to $\lambda_{1},\lambda_{2}\in R$ with λ_1_ ⋅ λ_2_ > 0. *FS*^−^ lies outside of the pyhsiological range due to *f*(*V*^−^, *x*^−^) > 1, an evaluation of the Jacobian of (14) yields conjugate-complex eigenvalues λ_1_, λ_2_ and hence a folded focus singularity.

The so-called singular strong canard *γ*_*s*_ is a particular trajectory of the desingularized system (14) that reaches FN on $S_{a}^{+}$ along the eigendirection *e*_1_ related to the strong eigenvalue λ_1_, where strongness follows from |λ_1_| > |λ_2_|. The singular strong canard *γ*_*s*_ can be calculated by a shooting method and is plotted in Fig 2. Likewise, the singular weak canard *γ*_*w*_ is associated with the eigendirection *e*_2_ corresponding to the weak eigenvalue λ_2_. the singular strong canard *γ*_*s*_ and the fold line *F*^+^ bound a region of trajectories that is referred to as the singular funnel. Inside the funnel, all trajectories approach the folded node FN along the weak eigendirection *e*_2_, see the dashed line in Fig 2.

The final key object of the (1, 2)-fast-slow analysis is the singular periodic orbit *s*_*o*_, that acts as a global return mechanism and is generated via a continuous concatenation of trajectories of the layer problem and the desingularized system. Starting at the folded node FN, the layer problem (15) is solved until the trajectory hits *P*(*F*^+^). From there, the desingularized system (14) is solved to continue the trajectory along $S_{a}^{-}$ until the fold line *F*^−^ is reached. The jump from *F*^−^ to *P*(*F*^−^) is obtained by again solving (15), after which the orbit is closed by returning the trajectory back to FN via the slow flow on $S_{a}^{+}$, see Fig 2. As will be discussed next, the decisive difference between Fig 2A and 2B is that the singular periodic orbit *s*_*o*_ lands within the funnel for *G*_*K*_ = 0.04 while it lands and stays outside of it for *G*_*K*_ = 0.06. That way the value of *G*_*K*_ will decide whether EADs occur or whether they do not.

#### EADs due to the folded node

**Fig 3:**
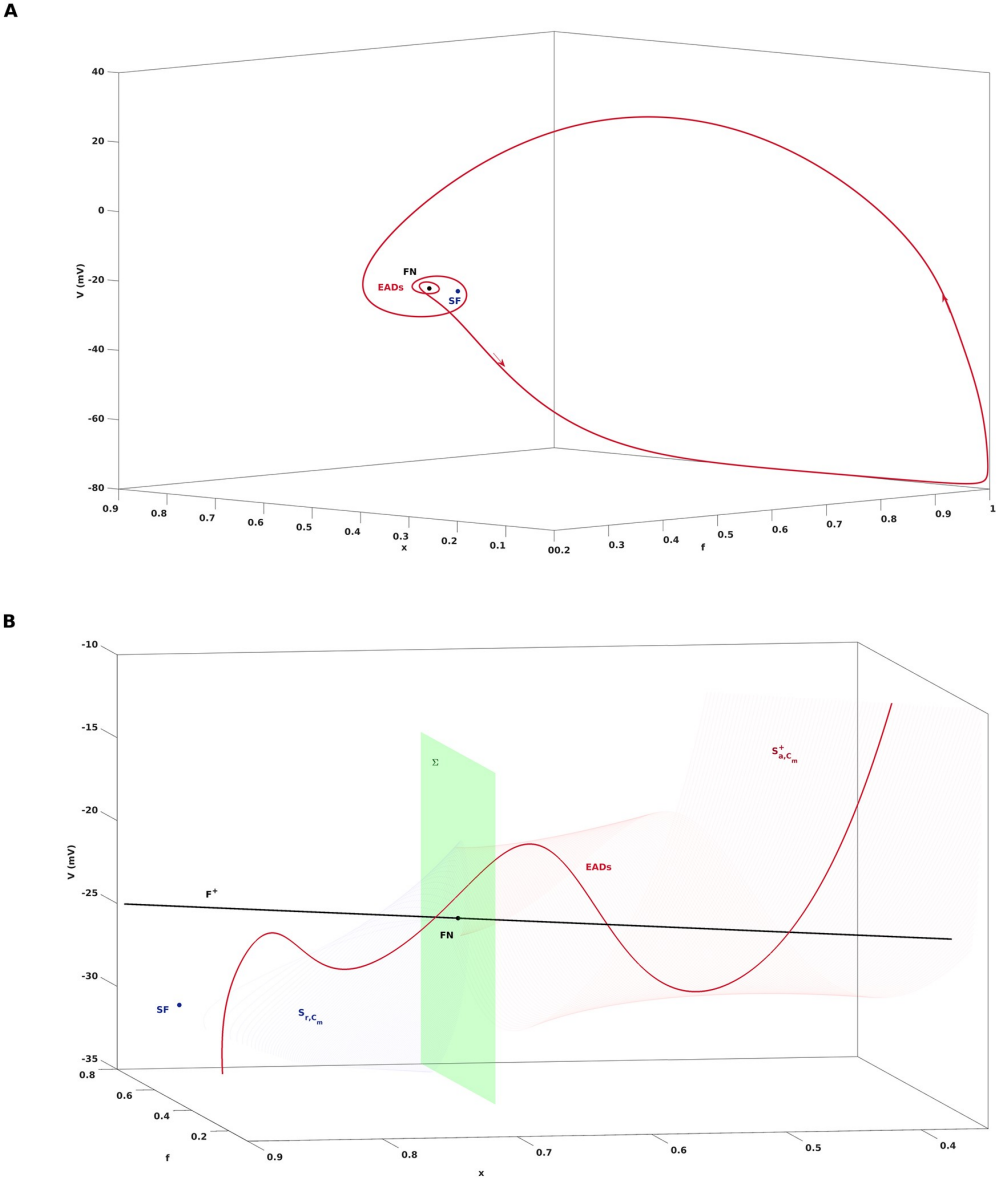
Twisted slow manifold near the folded node. A: Periodic orbit of (1) with *G*_*K*_ = 0.04 in phase space. The default parameter setting leads to an action potential with EADs in form of a 1^2^-MMO. B: The folded node causes a twisting of the slow manifolds and of the trajectory of (1) such that EADs occur. The attracting manifold $S_{a,C_{m}}^{+}$ and the repelling manifold $S_{r,C_{m}}$ were computed up to a surface *Σ* containing the folded node and transverse to the fold line *F*^+^. The red line is the projection of the trajectory from A.

The critical manifold *C*_0_ and the singular periodic orbit *s*_*o*_ are objects that are defined in the singular limit *C*_*m*_ → 0. The theory of MMOs with multiple time scales [21], [42] characterizes perturbations of these objects away from the singular limit, i.e., for *C*_*m*_ > 0, and allows us to draw conclusions about the dynamics of the full system (1) with *C*_*m*_ > 0. In particular, for *C*_*m*_ > 0 and away from the folded node FN, the critical manifold smoothly perturbs to a slow manifold with attracting manifolds $S_{a,C_{m}}^{+}$, $S_{a,C_{m}}^{-}$ and a repelling manifold $S_{r,C_{m}}$ according to geometric singular perturbation theory [43]. However, the theory of MMOs [44], [23], [24] implies that near the folded node FN, the critical manifold rather perturbs to twisted sheets. Fig 3B illustrates these twisted sheets in vicinity of the folded node of (14) for *G*_*K*_ = 0.04, which can be computed by continuation of solutions to boundary value problems associated with (1), see [24], [42]. Furthermore, the singular periodic orbit *s*_*o*_, if injected into the funnel, perturbs to a trajectory of the full system (1) that flows from $S_{a,C_{m}}^{+}$ to $S_{r,C_{m}}$ in a rotating manner, see Fig 3. That way, a folded node singularity, in combination with a suitable global return mechanism, may give rise to cardiac action potentials with EADs. If *s*_*o*_ passes by the funnel, as in case of *G*_*K*_ = 0.06, then *s*_*o*_ perturbs to a relaxation oscillation of type 1^0^, see Fig 1B, and a normal AP without EADs is obtained.

#### Parameter region of EADs occurence

**Fig 4:**
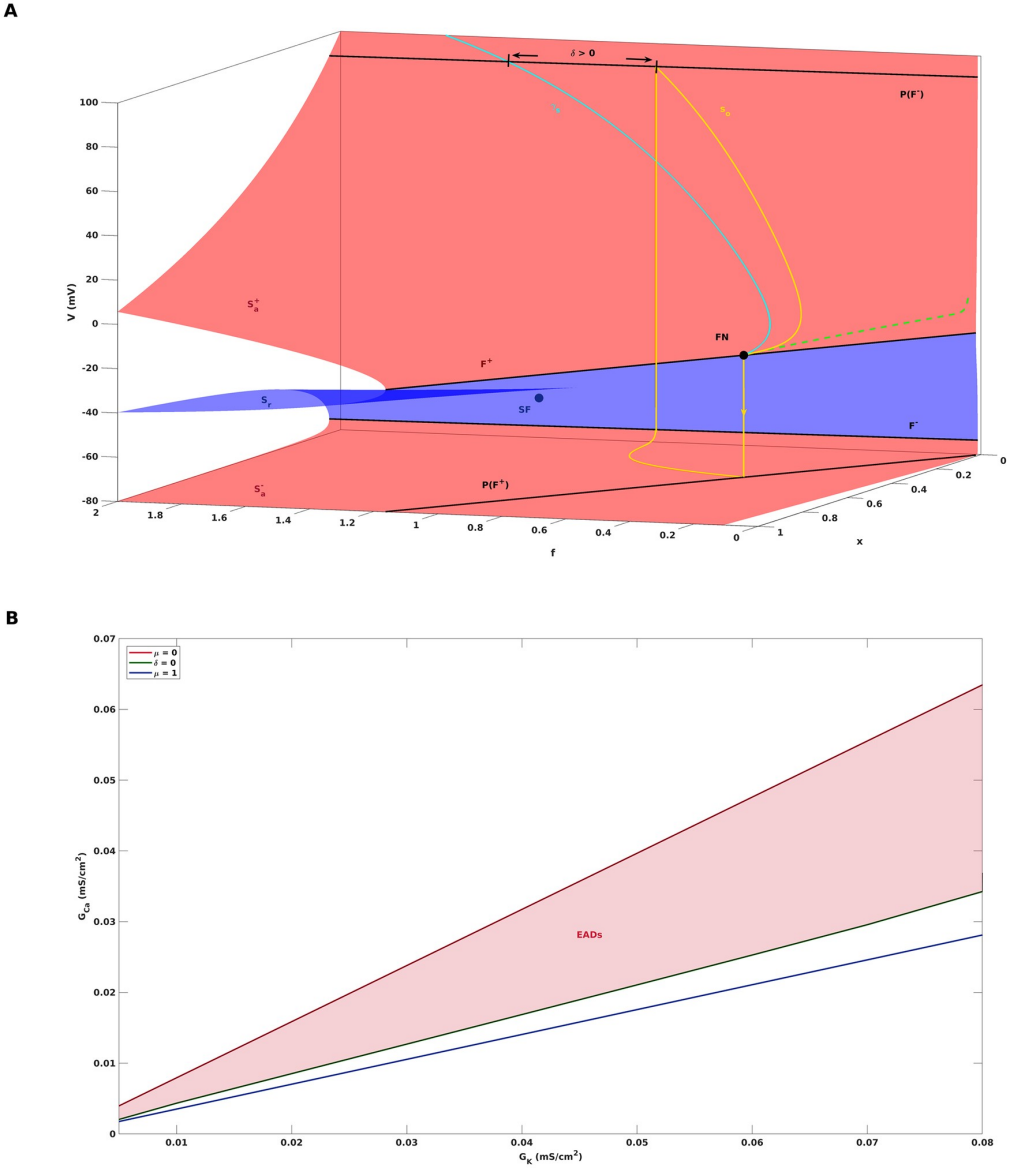
EADs boundaries according to folded node theory. A: The distance of the singular periodic orbit *s*_*o*_ from the singular strong canard *γ*_*s*_, measured along the projection *P*(*F*^−^) of the fold curve *F*^−^, is denoted by *δ*. For *δ* > 0 (as depicted), *s*_*o*_ enters the funnel and perturbs to an action potential with EADs away from the singular limit. B: Two-parameter bifurcation diagram predicting the region of parameter space for which EADs appear. The folded node singularity exists for 0 < *μ* < 1, according to folded node theory the occurence of EADs in addition requires *δ* > 0. For parameter combinations of *G*_*K*_ and *G*_*Ca*_ that lie above the curve *μ* = 0, the theory predicts convergence to a depolarized steady state. For parameter combinations of *G*_*K*_ and *G*_*Ca*_ that lie below the curve *δ* = 0, a relaxation oscillation of type 1^0^ is predicted.

If one denotes the ratio of the weak and strong eigenvalues by

$$\begin{array}{c} \mu =\frac{\lambda_{2}}{\lambda_{1}}, \end{array}$$

the condition for the existence of a folded node singularity can be formulated as 0 < *μ* < 1. In this notation, the folded node is either destroyed at *μ* = 0, where λ_2_ passes through 0 and the folded node turns into a folded saddle, or at *μ* = 1, where λ_1_ and λ_2_ coalesce and the folded node turns into a folded focus. The second condition for the occurence of EADs, i.e., the reinjection of *s*_*o*_ into the funnel, can be quantified in terms of the distance *δ* of *s*_*o*_ from the singular strong canard *γ*_*s*_. More precisely, *δ* measures the distance of the landing point of *s*_*o*_ on the projection *P*(*F*^−^) from *γ*_*s*_ along *P*(*F*^−^), see Fig 4 for an illustration of *δ*, and is positive if the landing point is inside of the funnel. Given these definitions of *μ* and *δ*, we constructed a bifurcation diagram that separates the (*G*_*K*_, *G*_*Ca*_)-parameter space into areas of different qualitative behaviour, see Fig 4. The region for which the folded node theory predicts the appearance of EADs is bounded by the curves defined by *μ* = 0 and *δ* = 0. Above the curve *μ* = 0, the theory predicts convergence to a depolarized steady state, while below the curve *δ* = 0, a relaxation oscillation of type 1^0^ is expected.

## Discussion

EADs in cardiac action potential models with different time scales may be considered as mixed mode oscillations of the form 1^*s*^. MMOs arise in a variety of scientific areas [25], [26], [27], [28], [29], and MMO theory [21], [42] discusses at least four different local mechanisms that give rise to such a behaviour: i) the tourbillon mechanism of a dynamic Hopf bifurcation, ii) a saddle focus equilibrium that goes through a singular Hopf bifurcation, iii) the passage through a folded node, and iv) three-time-scale problems with a singular Hopf bifurcation. Previous results of EADs analysis can either be linked with the tourbillon mechanism, see [17], [18], [20] for the case of a dynamic supercritical Hopf bifurcation and [20] for the case of a dynamic subcritical Hopf bifurcation, or with the saddle focus mechanism, see [20] and also the discussion below. Here, we for the first time have linked EADs with the folded node mechanism by showing that cardiac AP models may feature a folded node singularity in the desingularized slow subflow that is combined with a rejection of the singular periodic orbit *s*_*o*_ into the funnel area. Away from the singular limit, the critical manifold near the folded node perturbs to a twisted slow manifold, see Fig 3, and its twisting finally organizes the small amplitude oscillations observed as EADs in the trajectory of the full system. One conceptual difference between i) and iii) is that i) is based on the separation of a single slow variable from the full system while in iii) two slow variables are separated. In the example discussed in this paper, the (1, 2)-fast-slow-analysis explained the occurrence of EADs in the parameter transition from *G*_*K*_ = 0.06 to *G*_*K*_ = 0.04, while the alternative (2, 1)-fast-slow-analysis could not give a proper illumination.

### Overestimation of EADs region by folded node theory

**Fig 5:**
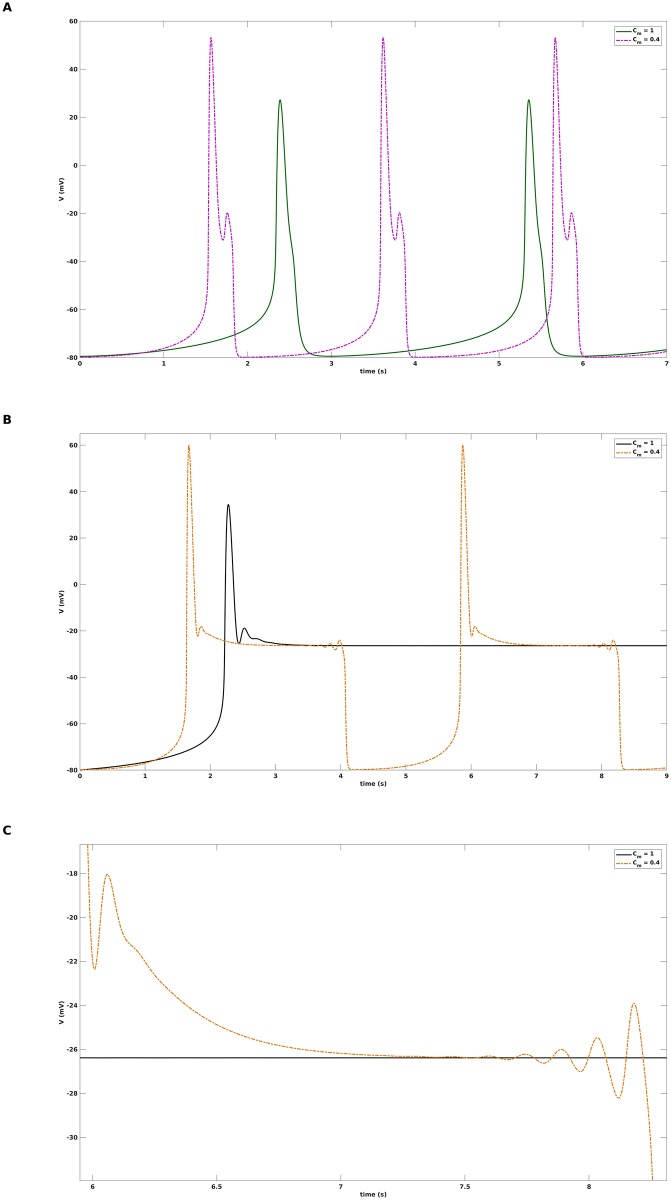
Predictive power of folded node theory depends on smallness of *C*_*m*_. The figure shows simulations of the full AP model (1) with different combinations of *G*_*K*_, *G*_*Ca*_ and *C*_*m*_. A: *G*_*K*_ = 0.05, *G*_*Ca*_ = 0.025. The point (0.05, 0.025) lies in the EADs area of Fig 4, still, for *C*_*m*_ = 1 one obtains an oscillation of the *l*^0^-pattern. However, for *C*_*m*_ = 0.04 one obtains EADs in accordance with the folded node theory. B: *G*_*K*_ = 0.034, *G*_*Ca*_ = 0.025. The point (0.034, 0.025) lies in the EADs area of Fig 4, still, for *C*_*m*_ = 1 the trajectory reaches a hyperpolarized steady state. However, for *C*_*m*_ = 0.04 one obtains EADs in accordance with the folded node theory. C: Zoom into EADs area of B, which shows a 1^9^-MMO pattern for *C*_*m*_ = 0.4.

One shortcoming of the (1, 2)-fast-slow-analysis is that the predictive power of singular perturbation theory is only guaranteed for *C*_*m*_ sufficiently small. While the sufficient smallness condition could be made slightly more precise in terms of $\sqrt{\varepsilon }⪡\mu$, see [21], with *ε* = *C*_*m*_/(*G*_*max*_ ⋅ *τ*_*f*_) obtained from a nondimensionalisation of (1), it turns out that the region of EADs occurence is overestimated in case of the default parameter value *C*_*m*_ = 1, see Fig 5 for an illustration based on simulations of the full AP model (1) with different parameter values. For example, both (*G*_*K*_, *G*_*Ca*_) = (0.05, 0.025) and (*G*_*K*_, *G*_*Ca*_) = (0.034, 0.025) lie inside of the shaded EADs region of Fig 4, which is based on the folded node theory, but a simulation of (1) with *C*_*m*_ = 1 leads to a 1^0^-oscillation corresponding to a normal action potential in the first case, and convergence to a hyperpolarized steady state in the second case. However, the simulations are in accordance with the prediction of Fig 4, if one reduces the capacitance to, e.g., *C*_*m*_ = 0.4, and action potentials with EADs of the form 1^1^ and 1^9^ are obtained. Note that in the singular limit *C*_*m*_ → 0, the trajectories converge to the singular periodic orbit *s*_*o*_, see Fig 2. Hence, as *C*_*m*_ is further reduced, the amplitudes of the small scale oscillations become smaller and smaller until they no longer can be numerically detected.

**Fig 6:**
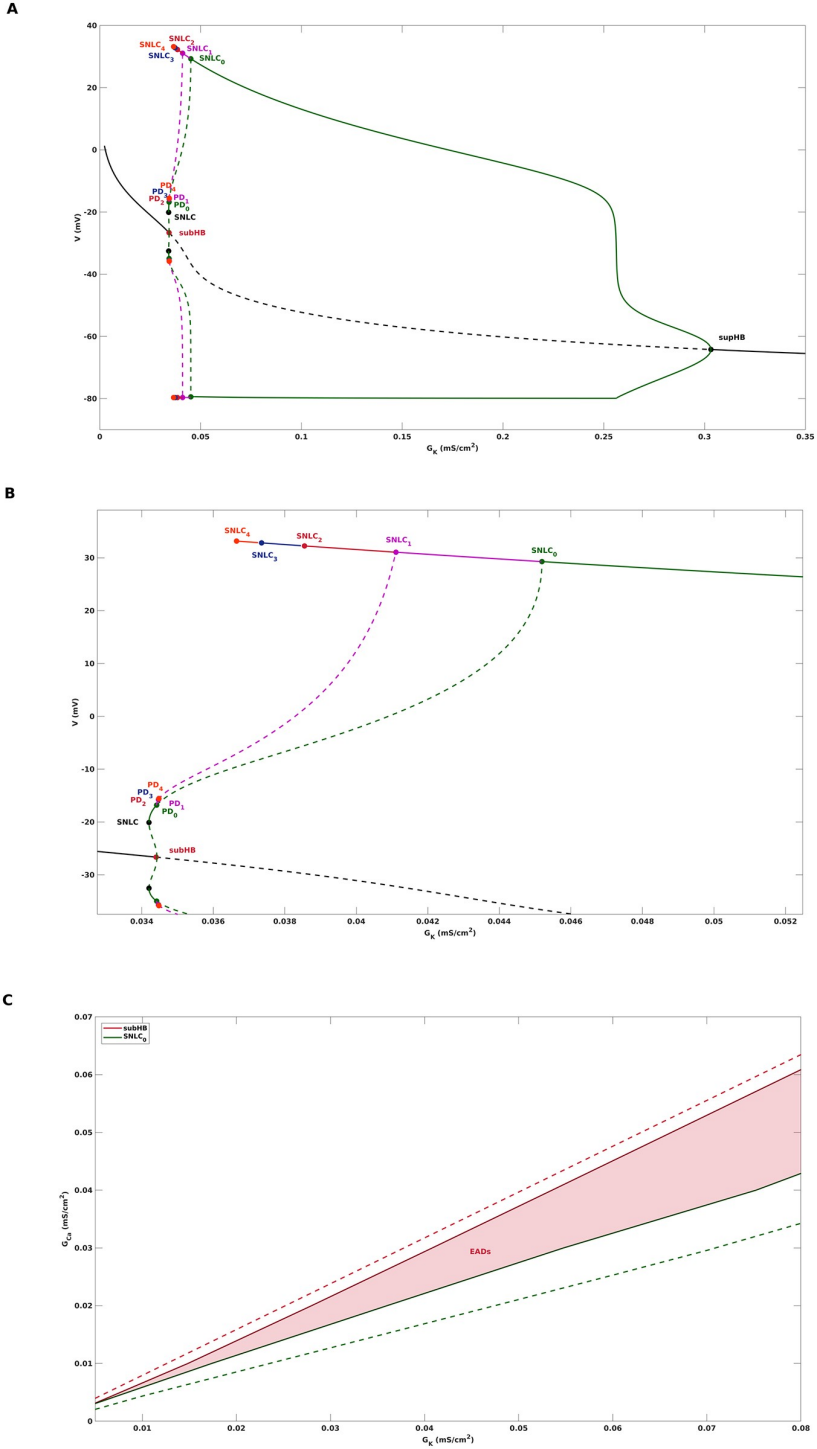
Actual EADs boundaries derived from full system analysis. A: Bifurcation diagram of the full system (1) with default parameter setting Table 1 and *G*_*K*_ as continuation parameter. Stable limit cycle oscillations of the type 1^0^ are born at the supercritical Hopf bifurcation supHB, turn into 1^*s*+1^-MMOs at the saddle node of limit cycle bifurcation SNLC_*s*_ and terminate at the subcritical Hopf bifurcation subHB. In particular, EADs occur in the *G*_*K*_-range between SNLC_0_ and subHB. B: Zoom into A. C: Two-parameter bifurcation diagram showing the region of parameter space for which EADs actually appear. For comparison, dashed lines depict the EADs boundaries predicted by the folded node theory, see Fig 4B.

In order to determine the actual EADs boundaries in the (*G*_*K*_, *G*_*Ca*_)-parameter space, we first performed a bifurcation analysis of the full system (1) with *G*_*K*_ as continuation parameter. Fig 6A shows the bifurcation diagram for the default parameter setting from Table 1 with *C*_*m*_ = 1. The curve corresponds to the equilibrium solutions of (1), which are unstable (dashed line) between the subcritical Hopf subHB and the supercritical Hopf bifurcation supHB. The coloured curves below and above represent the minimum and maximum voltages of stable (solid lines) and unstable (dashed lines) limit cycles of (1), which coalesce at saddle node of limit cycle bifurcations. Of particular interest is the sequence SNLC_*s*_, *s* = 0, 1, 2, …, as its members mark the transition from 1^*s*^ to 1^*s*+1^-MMOs, that is from APs with *s* EADs to *s* + 1 EADs, as *G*_*K*_ is reduced. For instance, the default setting *G*_*K*_ = 0.04 is located in the red area between SNLC_1_ and SNLC_2_ and hence features a 1^2^-limit cycle as shown in Fig 1. The unstable limit cycles that emerge from SNLC_*s*_ terminate at period doubling bifurcations of limit cycles PD_*s*_. While cascades of PDs often are associated with chaos, see [45] for an illustration of the PD-route to chaotic EADs dynamics, we did not observe any chaotic behaviour in the particular system at hand. Due to numerical difficulties, only the first two unstable limit cycle branches could be detected. Still, we conjecture that the branching pattern is repeated with ever smaller distances as *G*_*K*_ is decreased until the limit cycle behaviour finally terminates at subHB. Given that EADs appear in the *G*_*K*_-interval between SNLC_0_ and subHB, we next studied how that interval changes if the second channel conductance *G*_*Ca*_ is varied. Performing a sequence of curve continuations and tracking the locations of SNLC_0_ and subHB in the (*G*_*K*_, *G*_*Ca*_)-space gave rise to the region of EADs occurence shown in Fig 6C, which also visualizes the previously addressed overestimation obtained by the folded node theory.

### Distance to bifurcation as proarrhythmic risk metric

**Fig 7:**
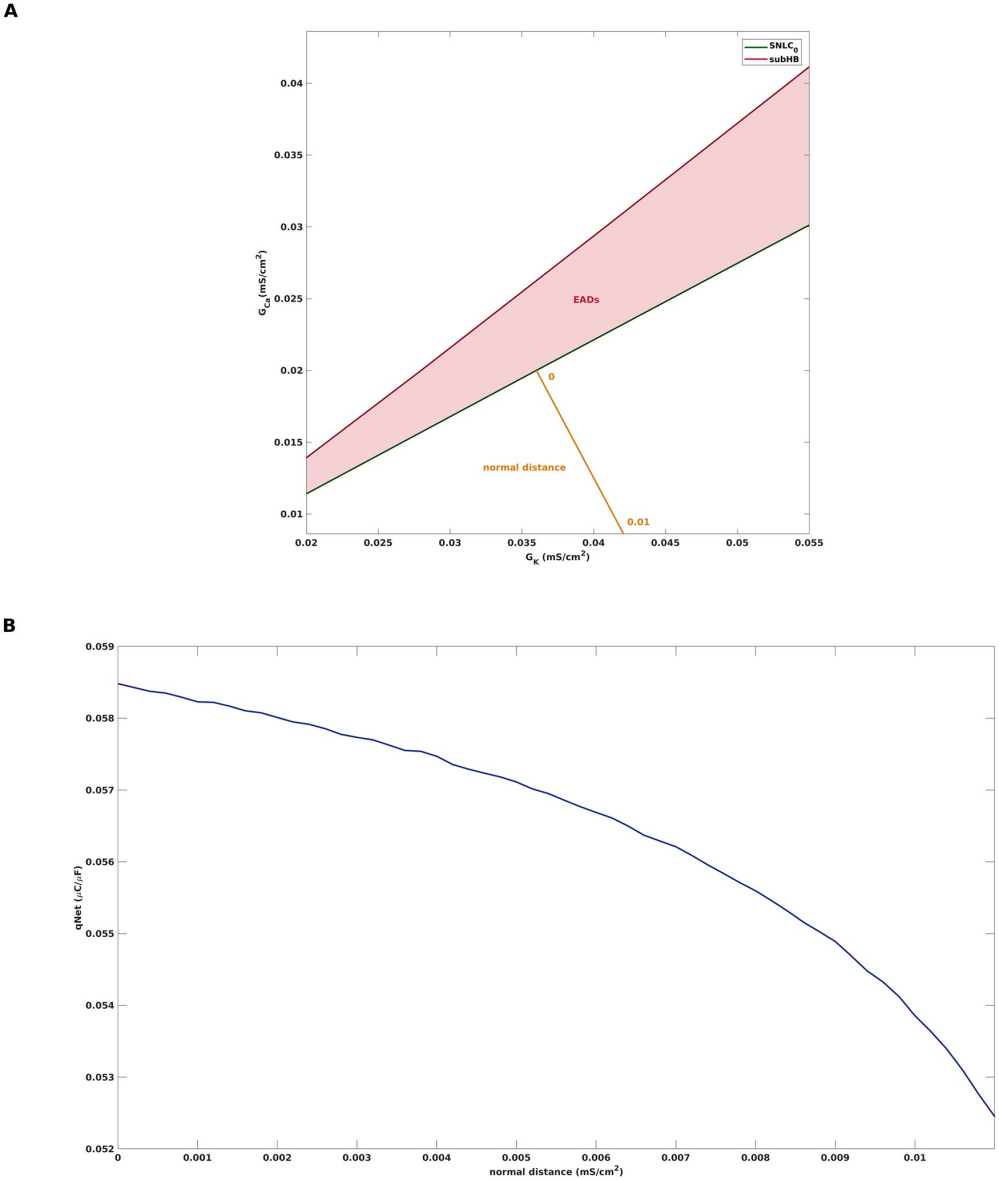
Normal distance to bifurcation as proarrhythmic risk metric. The figure illustrates the normal distance from the parameter region of EADs occurrence as a mechanism-based candidate measure of proarrhythmic risk. A: For instance, the AP system (1) with (*G*_*K*_, *G*_*Ca*_) = (0.042, 0.00835) is in normal distance 0.01 ms/cm^2^ from EADs occurrence. B: qNet computed with the AP model (1) is a monotonically decreasing function of the normal distance from SNLC_0_. The result is independent of the location chosen on the SNLC_0_-curve and in controversy to the qNet property found in [3].

One possible benefit of calculating the region of EADs behaviour from computational cardiac AP models is that distances from its boundaries could be used in quantifying the proarrhythmic risk of pharmaceutical compounds. Currently, the CiPA initiative (Comprehensive in vitro Proarrhythmic Assay) [4], [5] features the use of mathematical modelling of cardiac APs as part of future preclinical drug safety testing. Recently, a computational proarrhythmic risk metric called qNet was introduced in [3] and defined as the net charge carried by the key ionic currents integrated from the beginning to the end of the simulated AP beat. Using statistical classification algorithms, qNet was shown to correctly separate reference compounds into different risk categories, where drugs of high risk are linked to low values of qNet and drugs of low risk are linked to high values of qNet, see Fig 5 in [3]. Furthermore, qNet was found to correlate with the system’s robustness against EADs in the sense that EADs propensity is higher the lower the value of qNet. In our opinion, a manifest and mechanism-based measure of EADs propensity is the normal distance in parameter space from the SNLC_0_-bifurcation at which EADs behaviour starts, see Fig 7A. The closer the system is to the boundary, the higher is the proarrhythmic risk. Surprisingly, CiPA’s risk metric qNet, if computed with the AP model (1), is a monotonically decreasing function of the normal distance, see Fig 7B. This would associate lower proarrhythmic risk with lower values of qNet, which is in complete contrast to the association found in [3]. While this observation only constitutes a preliminary result that also might be influenced by the simplicity of (1), it still suggests a model dependency of the link between qNet and EADs propensity and calls for further investigations.

### Concurrence of folded node with saddle focus equilibrium

**Fig 8:**
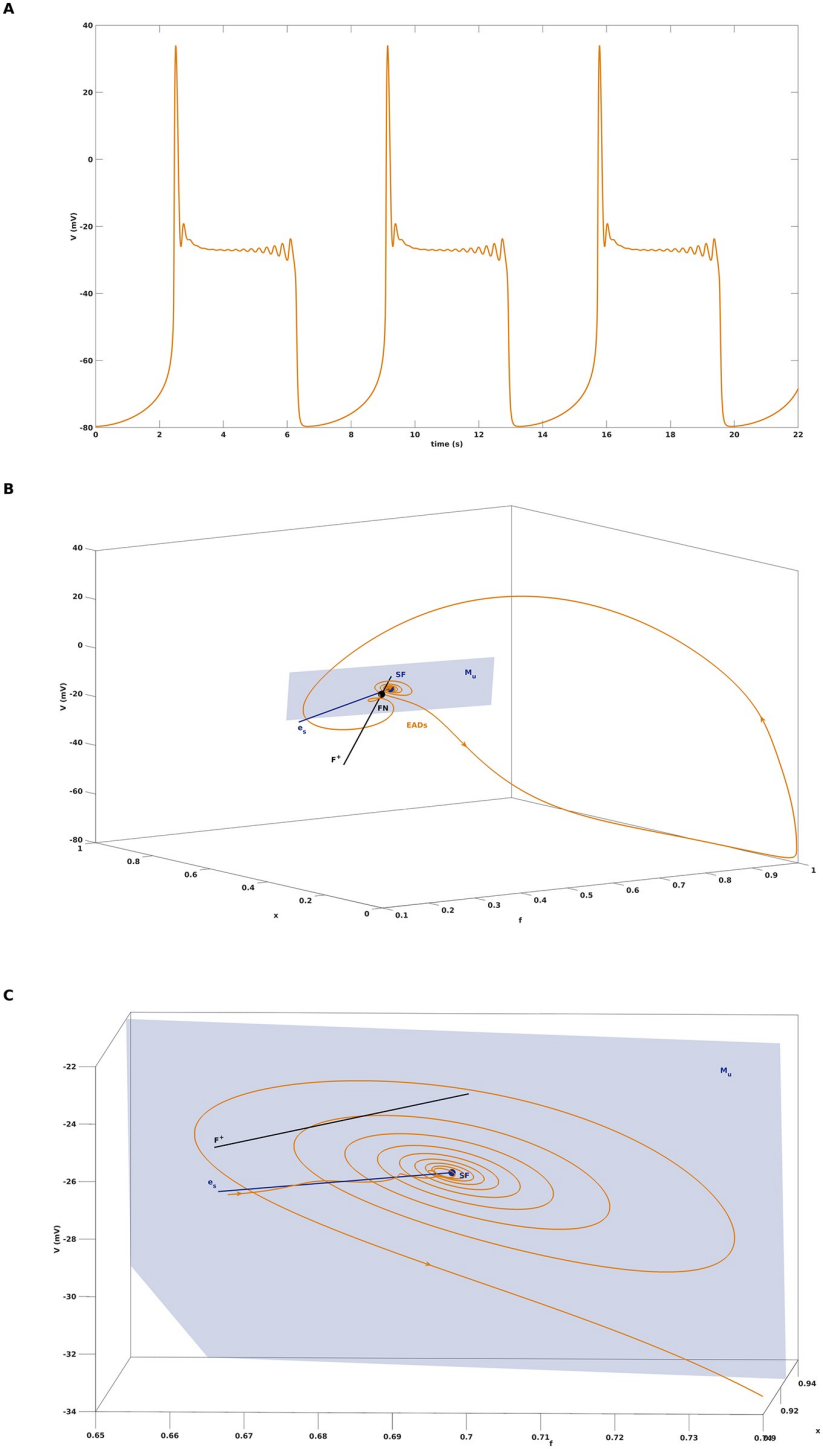
Concurrence of folded node with saddle focus equilibrium. A: An action potential with EADs in form of a 1^15^-MMO, obtained for the default parameter setting with *C*_*m*_ = 1 but *G*_*K*_ = 0.035. B: Visualization of the perodic orbit of A in the phase space. First, the EADs are organized by the twisted slow manifold due to the folded node FN, then EADs are caused by the spiraling along the unstable manifold of the saddle-focus equilibrium SF with tangential space *M*_*u*_. C: Zoom into B.

Finally, we return to Fig 5C and address the occurence of EADs in form of small scale oscillations with increasing amplitude. This behaviour is not an issue of the smallness of *C*_*m*_ but is also present for the default setting with *C*_*m*_ = 1 and, e.g., *G*_*K*_ = 0.035, see Fig 8A for a corresponding simulation of (1). While the first few oscillations are still organized by the twisted slow manifold associated with the folded node singularity FN, the growing oscillations are due to the saddle-focus equilibrium SF of the full system (1). More precisely, the trajectory approaches SF along its one-dimensional stable manifold, which is associated with the eigenvector *e*_*s*_ of the real eigenvalue λ_*s*_ < 0, and then spirals away along the unstable manifold with its tangential space *M*_*u*_ spanned by the pair of complex conjugate eigenvectors with positive real part, see Fig 8C. This mechanism has previously been related to the appearance of EADs in [20], but then independent of the folded node mechanism. As illustrated in Fig 8, the two mechanims may also coexist and consecutively shape the small scale oscillations, a phenomenon that was discussed in [21] in the context of the Koper model. Note that FN and SF also coexist for the parameter settings represented in Figs 2 and 3. However, the SF mechanism is only activated if the trajectory is properly conveyed to the SF after passage through the slow manifold, as exemplified in Fig 8B.

## Conclusion

In this paper, we have studied EADs in cardiac action potentials from the mathematical view point of mixed mode oscillations with multiple time scales. While the standard fast-slow analysis based on a single slow variable failed to explain the appearance of EADs in a parsimonious action potential model, a (1, 2)-fast-slow analysis based on two slow variables revealed that EADs may be caused by a folded node singularity if combined with a suitable global return mechanism. Furthermore, we have shown that the folded node coexists with a saddle focus equilibrium of the full system. Hence, EADs may result from the concurrence of two dynamical mechanisms, where the small scale oscillations first are induced by the twisted slow manifold associated with the folded node and then caused by spiraling along the unstable manifold of a saddle focus.

Our results form a novel contribution to EADs theory and may find application, e.g., in the context of preclinical cardiac drug safety testing. Both the MMO theory of folded nodes and a bifurcation analysis of the full system allow to compute regions in the ion channel conductance parameter space where EADs occur. Pharmacology data of a test compound such as IC_50_ values then defines its location in that space, and the normal distance from the boundary of the EADs region could be used as a mechanism based proarrhythmic risk metric. Interestingly since in contrast to [3], the normal distance associates EADs propensity with high values of the risk metric qNet, which is currently proposed by CiPA and defined based on statistical classification rather than on a EADs generating mechanism. In order to further investigate the suitability of the normal distance for preclinical cardiac safety testing, the concept needs to be tested by means of the the optimized IKr-dyn ORd model [3], which was used to define and validate the qNet risk metric. However, this in return requires to develop novel theoretical and computational tools for the MMO analysis of medium-to-large scale AP models.

State-of-the-art models [32], [9], [3], [12] comprise dozens of state variables and hundreds of model parameters. Hence, one limitation of our study is that we only dealt with a parsimonious cardiac action potential model, which in particular does not account for the sodium current, a known contributor to EADs [19]. However, it is the model’s simplicity that made it amenable to multiple time scale analysis [42], and the dynamical EADs mechanisms characterized by its help are likely to also be present in the more complex AP models. Future work will include the performance of tailored experiments with human induced pluripotent stem cell derived cardiomyocytes to challenge the EADs hypothesis generated in this paper. Furthermore, we aim to investigate whether a three-time-scale analysis of cardiac AP models can attribute EADs also with the remaining MMO-mechanism iv) of the listing given at the beginning of the discussion section.

## References

[1] Weiss JN, Garfinkel A, Karagueuzian HS, Chen PS, Qu Z. Early afterdepolarizations and cardiac arrhythmias. Heart Rhythm. 2010;7(12):1891–1899. 10.1016/j.hrthm.2010.09.017 20868774 PMC3005298

[2] Yan GX, Wu Y, Liu T, Wang J, Marinchak RA, Kowey PR. Phase 2 early afterdepolarization as a trigger of polymorphic ventricular tachycardia in acquired Long-QT syndrome. Circulation. 2001;103(23):2851–2856. 10.1161/01.CIR.103.23.2851 11401944

[3] Dutta S, Chang KC, Beattie KA, Sheng J, Tran PN, Wu WW, et al Optimization of an in silico cardiac cell model for proarrhythmia risk assessment. Frontiers in Physiology. 2017;8:616 10.3389/fphys.2017.00616 28878692 PMC5572155

[4] Sager PT, Gintant G, Turner JR, Pettit S, Stockbridge N. Rechanneling the cardiac proarrhythmia safety paradigm: a meeting report from the Cardiac Safety Research Consortium. American heart journal. 2014;167(3):292–300. 10.1016/j.ahj.2013.11.004 24576511

[5] Colatsky T, Fermini B, Gintant G, Pierson JB, Sager P, Sekino Y, et al The comprehensive in vitro proarrhythmia assay (CiPA) initiative—update on progress. Journal of Pharmacological and Toxicological Methods. 2016;81:15–20. 10.1016/j.vascn.2016.06.002 27282641

[6] Fermini B, Hancox JC, Abi-Gerges N, Bridgland-Taylor M, Chaudhary KW, Colatsky T, et al A new perspective in the field of cardiac safety testing through the comprehensive in vitro proarrhythmia assay paradigm. Journal of Biomolecular Screening. 2016;21(1):1–11. 10.1177/1087057115594589 26170255

[7] Fenton FH, Cherry EM. Models of cardiac cell. Scholarpedia. 2008;3(8):1868 10.4249/scholarpedia.1868

[8] Zeng J, Rudy Y. Early afterdepolarizations in cardiac myocytes: mechanism and rate dependence. Biophysical Journal. 1995;68(3):949–964. 10.1016/S0006-3495(95)80271-7 7538806 PMC1281819

[9] O’Hara T, Virág L, Varró A, Rudy Y. Simulation of the undiseased human cardiac ventricular action potential: model formulation and experimental validation. PLoS Comput Biol. 2011;7(5):e1002061 10.1371/journal.pcbi.1002061 21637795 PMC3102752

[10] Passini E, Britton OJ, Lu HR, Rohrbacher J, Hermans AN, Gallacher DJ, et al Human In silico drug trials demonstrate higher accuracy than animal models in predicting clinical pro-arrhythmic cardiotoxicity. Frontiers in Physiology. 2017;8:668 10.3389/fphys.2017.00668 28955244 PMC5601077

[11] McMillan B, Gavaghan DJ, Mirams GR. Early afterdepolarisation tendency as a simulated pro-arrhythmic risk indicator. Toxicol Res. 2017;6:912–921. 10.1039/C7TX00141J PMC5779076 29456831

[12] Paci M, Pölönen RP, Cori D, Penttinen K, Aalto-Setälä K, Severi S, et al Automatic optimization of an in silico model of human iPSC derived cardiomyocytes recapitulating calcium handling abnormalities. Frontiers in Physiology. 2018;9:709 10.3389/fphys.2018.00709 29997516 PMC6028769

[13] Sato D, Xie LH, Sovari AA, Tran DX, Morita N, Xie F, et al Synchronization of chaotic early afterdepolarizations in the genesis of cardiac arrhythmias. Proceedings of the National Academy of Sciences. 2009;106(9):2983–2988. 10.1073/pnas.0809148106 PMC2651322 19218447

[14] Zimik S, Vandersickel N, Nayak AR, Panfilov AV, Pandit R. A comparative study of early afterdepolarization-mediated fibrillation in two mathematical models for human ventricular cells. PLOS ONE. 2015;10(6):1–20. 10.1371/journal.pone.0130632 PMC4488347 26125185

[15] Vandersickel N, Van Nieuwenhuyse E, Seemann G, Panfilov AV. Spatial patterns of excitation at tissue and whole organ level due to early afterdepolarizations. Frontiers in Physiology. 2017;8:404 10.3389/fphys.2017.00404 28690545 PMC5479889

[16] Kurata Y, Tsumoto K, Hayashi K, Hisatome I, Tanida M, Kuda Y, et al Dynamical mechanisms of phase-2 early afterdepolarizations in human ventricular myocytes: insights from bifurcation analyses of two mathematical models. American Journal of Physiology-Heart and Circulatory Physiology. 2017;312(1):H106–H127. 10.1152/ajpheart.00115.2016 27836893

[17] Tran DX, Sato D, Yochelis A, Weiss JN, Garfinkel A, Qu Z. Bifurcation and chaos in a model of cardiac early afterdepolarizations. Phys Rev Lett. 2009;102(25):258103 10.1103/PhysRevLett.102.258103 19659123 PMC2726623

[18] Qu Z, Xie LH, Olcese R, Karagueuzian HS, Chen PS, Garfinkel A, et al Early afterdepolarizations in cardiac myocytes: beyond reduced repolarization reserve. Cardiovascular Research. 2013;99(1):6–15. 10.1093/cvr/cvt104 23619423 PMC3687754

[19] Sato D, Clancy CE, Bers DM. Dynamics of sodium current mediated early afterdepolarizations. Heliyon. 2017;3(9):e00388 10.1016/j.heliyon.2017.e00388 28924617 PMC5591396

[20] Kügler P. Early afterdepolarizations with growing amplitudes via delayed subcritical Hopf bifurcations and unstable manifolds of saddle foci in cardiac action potential dynamics. PLOS ONE. 2016;11(3):1–14. 10.1371/journal.pone.0151178 PMC4792449 26977805

[21] Desroches M, Guckenheimer J, Krauskopf B, Kuehn C, Osinga H, Wechselberger M. Mixed-mode oscillations with multiple time scales. SIAM Review. 2012;54(2):211–288. 10.1137/100791233

[22] Szmolyan P, Wechselberger M. Canards in ℝ^3^. Journal of Differential Equations. 2001;177(2):419—453. 10.1006/jdeq.2001.4001

[23] Brøns M, Krupa M, Weichselberger M. Mixed mode oscillations due to the generalized canard phenomenon. Fields Institute Communications. 2006;49:39–63.

[24] Desroches M, Krauskopf B, Osinga H. The geometry of slow manifolds near a folded node. SIAM Journal on Applied Dynamical Systems. 2008;7(4):1131–1162. 10.1137/070708810

[25] Vo T, Bertram R, Tabak J, Wechselberger M. Mixed mode oscillations as a mechanism for pseudo-plateau bursting. Journal of Computational Neuroscience. 2010;28(3):443–458. 10.1007/s10827-010-0226-7 20186476 PMC3151174

[26] Vo T, Bertram R, Wechselberger M. Bifurcations of canard-induced mixed mode oscillations in a pituitary Lactotroph model. Discrete & Continuous Dynamical Systems—A. 2012;32(8):2879–2912. 10.3934/dcds.2012.32.2879

[27] Bertram R, Tabak J, Teka W, Vo T, Wechselberger M, Kirk V, et al Mathematical Analysis of Complex Cellular Activity Frontiers in Applied Dynamical Systems: Reviews and Tutorials. Springer International Publishing; 2015.

[28] Roberts A, Widiasih E, Wechselberger M, Jones CKRT. Mixed mode oscillations in a conceptual climate model. Physica D: Nonlinear Phenomena. 2015;292-293:70–83. 10.1016/j.physd.2014.11.003

[29] Roberts A, Guckenheimer J, Widiasih E, Timmermann A, Jones C. Mixed-mode oscillations of El Nino–southern oscillation. Journal of the Atmospheric Sciences. 2016;73(4):1755–1766. 10.1175/JAS-D-15-0191.1

[30] Noble D. A modification of the Hodgkin-Huxley equations applicable to Purkinje fibre action and pacemaker potentials. The Journal of Physiology. 1962;160(2):317–352. 10.1113/jphysiol.1962.sp006849 14480151 PMC1359535

[31] Beeler GW, Reuter H. Reconstruction of the action potential of ventricular myocardial fibres. The Journal of Physiology. 1977;268(1):177–210. 10.1113/jphysiol.1977.sp011853 874889 PMC1283659

[32] ten Tusscher KH, Noble D, Noble PJ, Panfilov AV. A model for human ventricular tissue. American Journal of Physiology-Heart and Circulatory Physiology. 2004;286(4):H1573–H1589. 10.1152/ajpheart.00794.2003 14656705

[33] Fenton F, Karma A. Vortex dynamics in three-dimensional continuous myocardium with fiber rotation: Filament instability and fibrillation. Chaos: An Interdisciplinary Journal of Nonlinear Science. 1998;8(1):20–47. 10.1063/1.166311 12779708

[34] Mitchell CC, Schaeffer DG. A two-current model for the dynamics of cardiac membrane. Bulletin of Mathematical Biology. 2003;65(5):767–793. 10.1016/S0092-8240(03)00041-7 12909250

[35] Bueno-Orovio A, Cherry EM, Fenton FH. Minimal model for human ventricular action potentials in tissue. Journal of Theoretical Biology. 2008;253(3):544—560. 10.1016/j.jtbi.2008.03.029 18495166

[36] Gray RA, Pathmanathan P. A parsimonious model of the rabbit action potential elucidates the minimal physiological requirements for alternans and spiral wave breakup. PLOS Computational Biology. 2016;12(10):1–21. 10.1371/journal.pcbi.1005087 PMC5066986 27749895

[37] Sato D, Xie LH, Nguyen TP, Weiss JN, Qu Z. Irregularly appearing early afterdepolarizations in cardiac myocytes: random fluctuations or dynamical chaos? Biophysical Journal. 2010;99(3):765—773. 10.1016/j.bpj.2010.05.019 20682253 PMC2913181

[38] Xie Y, Izu LT, Bers DM, Sato D. Arrhythmogenic transient dynamics in cardiac myocytes. Biophysical Journal. 2014;106(6):1391—1397. 10.1016/j.bpj.2013.12.050 24655514 PMC3984988

[39] MATLAB Release 2017b; 2017.

[40] Dhooge A, Govaerts W, Kuznetsov YA. matcont: A matlab package for numerical bifurcation analysis of ODEs. ACM TOMS. 2003;29:141–164. 10.1145/779359.779362

[41] Doedel EJ, Fairgrieve TF, Sandstede B, Champneys AR, Kuznetsov YA, Wang X. AUTO-07P: Continuation and bifurcation software for ordinary differential equations; 2007.

[42] Kuehn C. Multiple Time Scale Dynamics Applied Mathematical Sciences. Springer International Publishing; 2015.

[43] Fenichel N. Geometric singular perturbation theory for ordinary differential equations. Journal of Differential Equations. 1979;31(1):53—98. 10.1016/0022-0396(79)90152-9

[44] Wechselberger M. Existence and bifurcation of canards in ℝ^3^ in the case of a folded node. SIAM Journal on Applied Dynamical Systems. 2005;4(1):101–139. 10.1137/030601995

[45] Kügler P, Bulelzai MAK, Erhardt AH. Period doubling cascades of limit cycles in cardiac action potential models as precursors to chaotic early afterdepolarizations. BMC Systems Biology. 2017;11(1):42 10.1186/s12918-017-0422-4 28376924 PMC5379775

